# The complete mitochondrial genome of the firefly *Aquatica hydrophila* (Jeng, Lai & Yang, 2003) (Coleoptera, Lampyridae) and its phylogenetic analysis

**DOI:** 10.1080/23802359.2025.2602245

**Published:** 2025-12-11

**Authors:** Xiao-Hua Guo, Jun Zhang, You-Jun Wu, Jiao-Meng Deng, Xiao-Li Fan, Zi-Long Zhong

**Affiliations:** a College of Ecology, Lishui University, Lishui, China; b Ecological Forestry Development Center of Suichang County, Lishui, China; c Forestry Technology Extension Station of Sanmen County, Taizhou, China

**Keywords:** Firefly, East Asian region, conservation genetics, mitochondrial DNA

## Abstract

The complete mitochondrial genome of *Aquatica hydrophila* spans 16,394 bp and includes the standard set of 37 genes, consisting of 13 protein-coding genes (PCGs), 22 tRNA genes, and 2 rRNA genes, along with a control region. All PCGs initiate with the typical ATN start codon except *nd1*; most terminate with TAA/TAG, while *cox2*, *cox3*, *nd4*, and *nd5* use a single-T incomplete stop. The rRNA genes measure 1337 bp (*rrn16*) and 837 bp (*rrn12*). The control region is 2,219 bp, located between *rrn12* and *trnI*. Maximum-likelihood analysis of 13 concatenated PCGs positions *A. hydrophila* within a monophyletic *Aquatica*, as a sister taxon to (*A. wuhana* + (*A. leii* + *A. ficta*)); *Aquatica* is recovered as sister to *Abscondita* with strong support. Together, these data provide a genomic reference for *A. hydrophila* that will facilitate future studies on lucioline phylogeny, species delimitation, and the conservation of aquatic firefly populations. Because *Aquatica* species are specialists of clean, flowing freshwater habitats, the new mitogenome of *A. hydrophila* will also facilitate assessments of population connectivity and support conservation planning for aquatic fireflies in East Asia.

## Introduction

*Aquatica hydrophila* (Jeng et al. 2003) is an East Asian aquatic-larval firefly, first described from Taiwan as *Luciola hydrophila* and later transferred to *Aquatica*, a lucioline genus with obligately aquatic larvae (e.g. *A. lateralis, A. ficta, A. leii, A. wuhana*; Fu et al. 2010; Jeng et al. 2003). Recent morphology + mitogenome work recognizes seven *Aquatica* species, confirms monophyly, and places the genus sister to *Nipponoluciola* (Fu et al. 2024). Larvae inhabit streams or paddies, feed benthically and possess lateral abdominal gills, and adults show species-specific courtship flashes—underscoring the need for denser genomic sampling to refine species boundaries and intergeneric relationships in the subfamily Luciolinae.

Mitochondrial genomes, compact (typically ∼16–17 kb) and conserved in gene content, are widely used for beetle systematics, DNA barcoding, and population genetics, and also provide characters for comparative genomics (Cameron 2014). In Luciolinae, complete mitogenomes have been published for some congeners or close relatives like *A. leii* and *A. lateralis*, but species - level coverage in *Aquatica* is incomplete (Jiao et al. 2015; Maeda et al. 2017a). Besides mitochondrial data, nuclear genomes of luminous beetles (including *A. lateralis*) have revealed broader bioluminescence evolutionary patterns, which further encourages using mitogenome resources for firefly phylogenetic inference (Fallon et al. 2018).

Here we assemble and annotate the complete mitochondrial genome of *A. hydrophila* and infer its placement within Luciolinae using protein-coding genes (PCGs), providing a comparative resource for taxonomy, phylogeny, and conservation genetics of aquatic fireflies.

## Materials and methods

**Figure 1.**
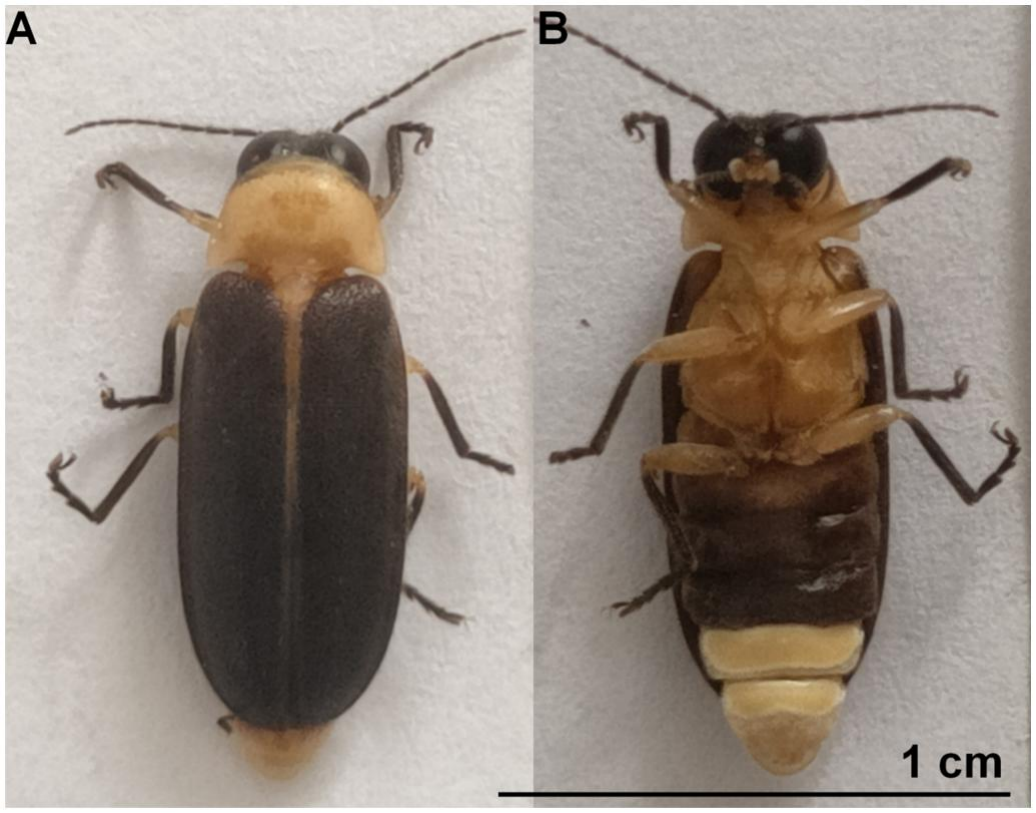
Reference image of *Aquatica hydrophila*. (A) Dorsal view. (B) Ventral view. Both photographs were taken by the author of this article, Xiao-Hua Guo.

In June 2024, adult *A. hydrophila* were collected in Baishanzu National Park, Lishui, Zhejiang, China (27°45′39.60″N, 119°11′52.80″E). Identification followed Jeng et al. (2003) and Fu et al. (2010) based on males with weakly serrate antennae, a translucent yellow-orange pronotum with a median dark stripe, uniformly brown elytra lacking pale tips, paired light organs on sternites VI–VII, and slight sexual dimorphism (males 8–10 mm; females larger). Specimens were photographed (Nikon D850), briefly chilled, and thoracic flight muscle preserved in 95% ethanol. Vouchers LSU-ZJ2024-06-003 (Figure 1) are deposited at the College of Ecology, Lishui University (contact: Xiao-Li Fan, Fanlilly@163.com).

Genomic DNA was extracted from thoracic muscle (Sangon Rapid Animal kit). A 350-bp TruSeq Nano library was sequenced on an Illumina HiSeq 2500 (PE150), yielding 7.80 Gb raw and 7.64 Gb clean data after quality filtering with fastp v0.20.0 (Chen et al. 2018), removing reads containing adapter contamination, reads with >10% ambiguous bases (N), and reads in which >50% of bases had a Phred quality score < Q20. Reads were mapped to the *A. wuhana* mitogenome (GenBank accession No.: KX758086) with BWA-MEM v0.7.17 (Li 2013), and the mapped set assembled with SPAdes v4.10 (Prjibelski et al. 2020) to a circular contig. To check reference bias, the same reads were assembled de novo with GetOrganelle v1.7.7 (Jin et al. 2020; k-mers 21–127), producing the same 16,394-bp contig (37 genes plus control region). The genome was circularized with MitoZ v2.4 (Meng et al. 2019) and polished twice with Pilon v1.24 (Walker et al. 2014). Annotation used MITOS2 (Donath et al. 2019) for initial prediction of mitochondrial PCGs, rRNAs, and tRNAs, corroborated by BLAST+ v2.28 (Camacho et al. 2009) to confirm gene identities and boundaries against published mitogenomes, MiTFi (Jühling et al. 2012) for tRNA detection, and Infernal v1.1 (Nawrocki and Eddy 2013) for the identification of structured RNAs; annotations were then refined in Geneious Prime v.2024.0.7 (Geneious 2025). A circular mitochondrial map was generated in Proksee (Grant et al. 2023). Coverage was validated by Bowtie2 v2.3.4 (Langmead and Salzberg 2012); per-base depth was computed with SAMtools v1.16.1 (Li et al. 2009) and plotted with ggplot2 (Wickham 2016; Fig. S1).

For phylogeny, we analyzed 11 mitogenomes, including our *A. hydrophila* and 10 previously published lampyrid mitogenomes from GenBank for *Pyrocoelia praetexta*, *P. thibetana*, *Lampyris noctiluca*, *Diaphanes citrinus*, *Photinus pyralis*, *Aquatica ficta*, *A. lateralis*, *A. wuhana*, *A. leii*, and *Abscondita anceyi* (accession numbers in Table S1), with *Rhagophthalmus ohbai* as outgroup. The 13 PCGs were extracted in PhyloSuite v1.2.1 (Zhang et al. 2020), codon-aligned with MAFFT v7.388 (Katoh and Standley 2013), and concatenated. ML trees were inferred in IQ-TREE v2.1.2 (Minh et al. 2020) under the ModelFinder-selected (Kalyaanamoorthy et al. 2017) GTR+F + I + G4 model, with 1,000 bootstrap replicates. The tree was visualized/annotated in iTOL v6 (Letunic and Bork 2021).

## Results

**Figure 2.**
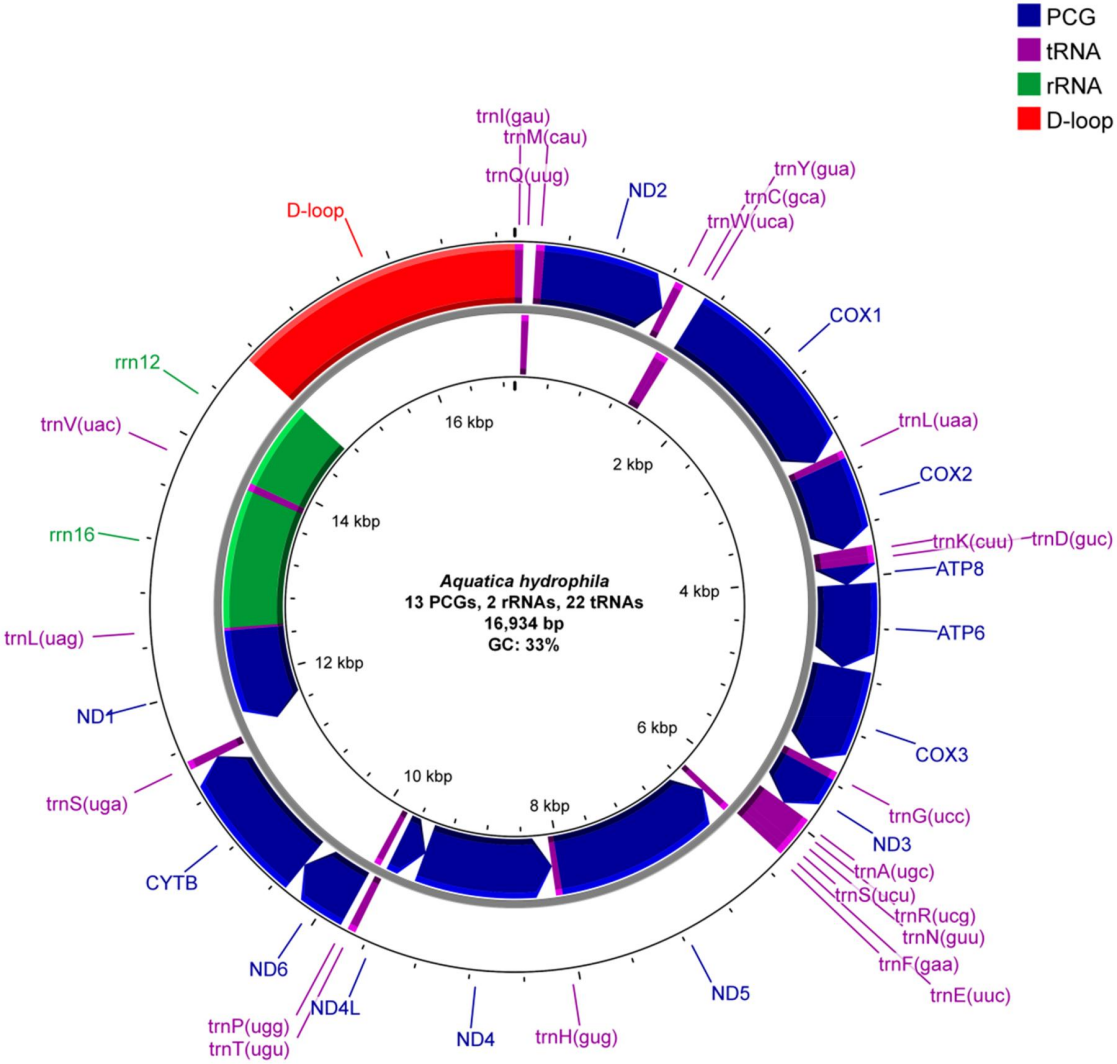
Circular map of the *Aquatica hydrophila* mitochondrial genome. Arrows indicate transcriptional directions.

The *A. hydrophila* mitogenome is 16,394 bp, with 37 genes (13 PCGs, 22 tRNAs, 2 rRNAs) plus a control region. Nucleotide composition: A 42.09%, T 32.93%, G 9.12%, C 15.86% (GC 24.98%). The majority strand carries 23 genes (9 PCGs, 14 tRNAs); the minority strand carries 14 (4 PCGs, 8 tRNAs, 2 rRNAs). PCGs start with ATN (except *nd1*) and end with TAA/TAG, except *cox2*, *cox3*, *nd4*, *nd5*, which use a single T as an incomplete stop. tRNAs are 59–70 bp. *rrn16* and *rrn12* are 1,337 bp and 837 bp (GC 18.77% and 18.76%). The control region is 2,219 bp (GC 17.71%) between *rrn12* and *trnI* (Figure 2).

**Figure 3.**
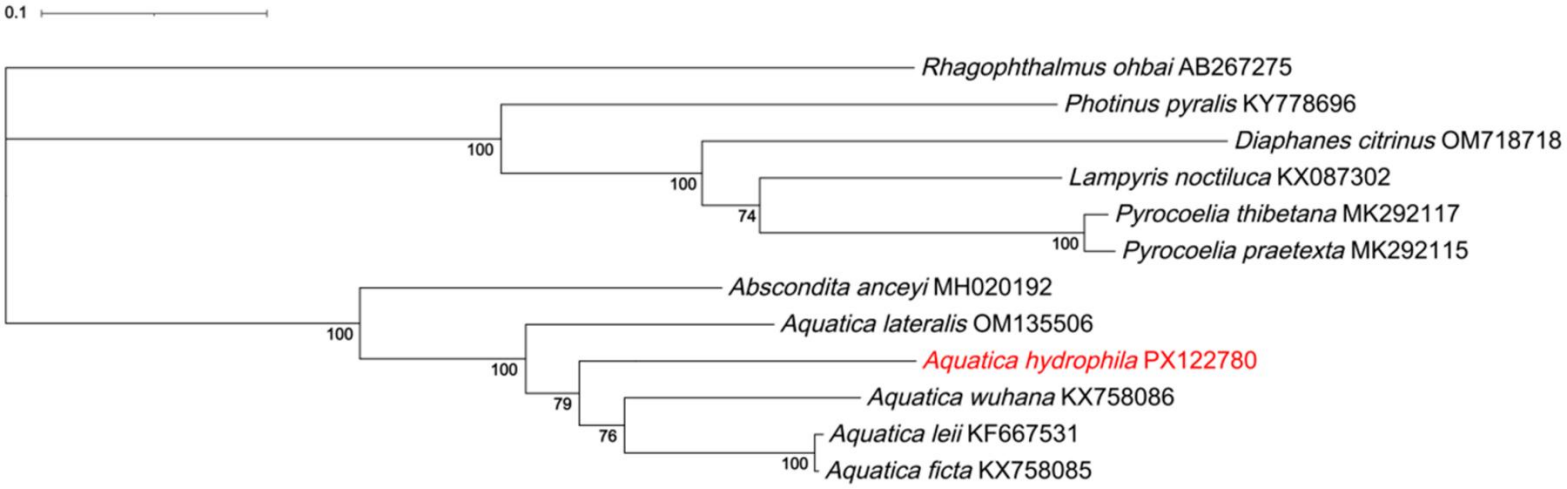
Maximum-likelihood phylogenetic tree based on the whole mitogenomes of *Aquatica hydrophila* and 10 species of Lampyridae family. Bootstrap values >70% are displayed above the branches. GenBank accession numbers for the mitogenomic sequences of all species are shown in Table S1.

In the ML tree based on 13 mitochondrial PCGs (Figure 3), *A. anceyi* formed a strongly supported clade with *Aquatica* (bootstrap = 100). Within *Aquatica*, *A. lateralis* was recovered as sister to the remaining four species with maximal support (bootstrap = 100). *A. hydrophila* then grouped with the clade comprising *A. wuhana*, *A. leii* and *A. ficta* with moderate support (bootstrap = 79), and within this clade *A. wuhana* was sister to the pair *A. leii* + *A. ficta* (bootstrap = 76). The sister relationship between *A. leii* and *A. ficta* was strongly supported (bootstrap = 100).

## Discussion and conclusion

The *A. hydrophila* mitogenome (16,394 bp) retains the canonical 37 genes (13 PCGs, 22 tRNAs, 2 rRNAs) and ancestral order, including the IQM tRNA cluster and *rrn12*–control region–*trnI*. Composition is strongly A + T-biased (∼75%), and four PCGs (*cox2*, *cox3*, *nd4*, *nd5*) end with incomplete stop codons—both typical of Luciolinae. The control region is 2219 bp, long for the subfamily yet within reported ranges. Metrics match congeners such as *A. leii* and *Luciola cruciata* (Cameron 2014; Jiao et al. 2015; Maeda et al. 2017b).

Our ML analysis of 13 concatenated PCGs nests *A. hydrophila* within a well-supported *Aquatica*; it is sister to (*A. wuhana* + (*A. leii* + *A. ficta*)). *Aquatica* is monophyletic and sister to *Abscondita*, consistent with recent mitogenome studies (Jiao et al. 2015; Hu and Fu 2018; Chen et al. 2019). Studies with denser lucioline sampling sometimes place *Aquatica* near *Nipponoluciola* (Fu et al. 2010, 2024); our *Abscondita* sister result likely reflects mtDNA-only data and limited sampling. This limited coverage mainly reflects the small number of complete, reliably annotated mitogenomes that were available in public databases for *Aquatica* and closely related lucioline taxa at the time of our analysis. As additional complete mitogenomes and nuclear loci become available—ideally including *Nipponoluciola*—broader taxon sampling will be needed to test these relationships and to clarify patterns among aquatic-larval lineages (Fallon et al. 2018; Fu et al. 2024).

We present the first complete mitogenome of *A. hydrophila* (16.4 kb) with standard genes, ancestral order, strong A + T bias, and an extended control region. A 13-PCG ML tree nests it within *Aquatica*, sister to (*A. wuhana* + (*A. leii* + *A. ficta*)), and recovers *Aquatica* as monophyletic and sister to *Abscondita*. This genome serves as a vetted reference for Luciolinae systematics and conservation and a basis for broader (mito)phylogenomic sampling in *Aquatica*.

## Supplementary Material

Figure S1 and table s1.doc

## Data Availability

The genome sequence data supporting this study are openly available in GenBank of NCBI at https://www.ncbi.nlm.nih.gov under the accession number PX122780. The associated BioProject, SRA, and Biosample numbers are PRJNA1303815, SRR34931746, and SAMN50536482, respectively.
