# Supplementary material for: The complete mitochondrial genome of the firefly Aquatica hydrophila (Jeng, Lai & Yang, 2003) (Coleoptera, Lampyridae) and its phylogenetic analysis
Source: Mitochondrial DNA B Resour. 2025 Dec 11;11(1):39–43. doi: 10.1080/23802359.2025.2602245 (PMC12704116; doi:10.1080/23802359.2025.2602245)
Supplement: Figure S1 and table s1.doc [file TMDN_A_2602245_SM0772.doc]

**The complete mitochondrial genome of the firefly *Aquatica hydrophila* (Coleoptera, Lampyridae) and its phylogenetic analysis**

Xiao-Hua Guo, Jun Zhang, You-Jun Wu, Jiao-Meng Deng, Xiao-Li Fan, Zi-Long Zhong


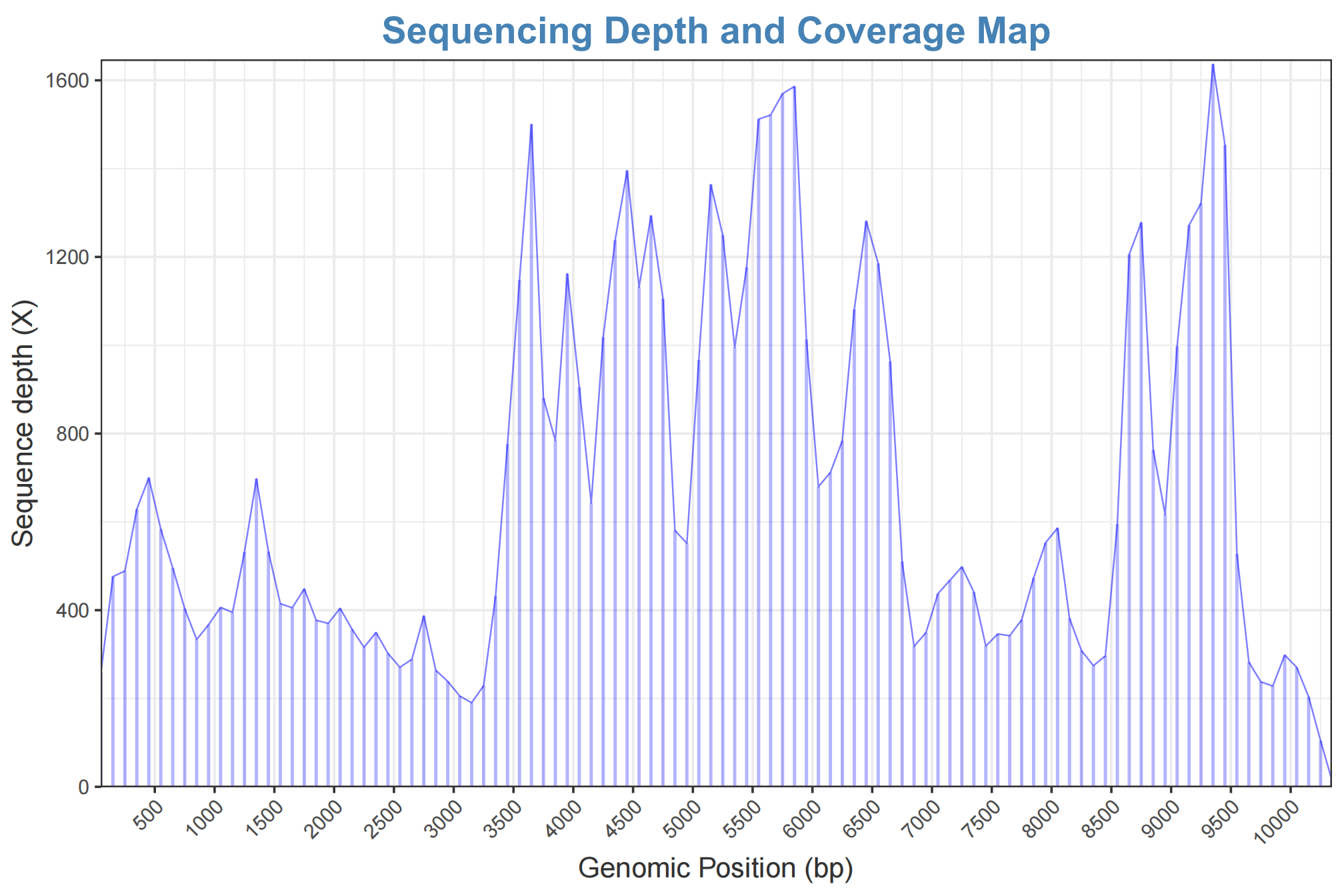


**Figure S1.** Coverage depth across the circular mitochondrial genome of *Aquatica hydrophila*. Illumina reads were mapped back to the final assembly, yielding a mean coverage depth of 479.78 × with uniform coverage (100% of sites covered). X and Y axis present nucleotide position of *A. hydrophila* mitochondrial genome and coverage depth, respectively.

Table S1. Mitochondrial genomes used for phylogenetic analysis of *Aquatica hydrophila.*

| **Taxon** | **Family/subfamily** | **GenBank accession** | **Sequence length**  **(bp)** | **Reference** |
| --- | --- | --- | --- | --- |
| *Pyrocoelia praetexta* | Lampyridae/Luciolinae | MK292115 | 17,634 | Chen et al., 2019 |
| *Pyrocoelia thibetana* | Lampyridae/Luciolinae | MK292117 | 18,054 | Chen et al., 2019 |
| *Lampyris noctiluca* | Lampyridae/Lampyrinae | KX087302 | 17,221 | unpublished |
| *Diaphanes citrinus* | Lampyridae/Luciolinae | MH651351 | 18,594 | Yang & Fu, 2019 |
| *Photinus pyralis* | Lampyridae/Lampyrinae | KY778696 | 17,081 | Fallon et al., 2018 |
| *Aquatica ficta* | Lampyridae/Luciolinae | KX758085 | 16,836 | Wang et al., 2017 |
| *Aquatica lateralis* | Lampyridae/Luciolinae | OM135506 | 16,851 | unpublished |
| *Aquatica wuhana* | Lampyridae/Luciolinae | KX758086 | 16,186 | Wang et al., 2017 |
| *Aquatica hydrophila* | Lampyridae/Luciolinae | PX122780 | 16,394 | this study |
| *Aquatica leii* | Lampyridae/Luciolinae | KF667531 | 16,856 | unpublished |
| *Abscondita anceyi* | Lampyridae/Luciolinae | MH020192 | 16,519 | Hu & Fu, 2018 |
| *Rhagophthalmus ohbai* | Rhagophthalmidae/Rhagophthalminae | AB267275 | 15,704 | Li et al., 2007 |
